# Testing and Treating Women after Unsuccessful Conservative Treatments for Overactive Bladder or Mixed Urinary Incontinence: A Model-Based Economic Evaluation Based on the BUS Study

**DOI:** 10.1371/journal.pone.0160351

**Published:** 2016-08-11

**Authors:** Ilias Goranitis, Pelham Barton, Lee J. Middleton, Jonathan J. Deeks, Jane P. Daniels, Pallavi Latthe, Arri Coomarasamy, Suneetha Rachaneni, Shanteela McCooty, Tina S. Verghese, Tracy E. Roberts

**Affiliations:** 1 Health Economics Unit, University of Birmingham, Birmingham, United Kingdom; 2 Birmingham Clinical Trials Unit, University of Birmingham, Birmingham, United Kingdom; 3 Public Health, Epidemiology and Biostatistics, University of Birmingham, Birmingham, United Kingdom; 4 School of Clinical and Experimental Medicine, University of Birmingham, Birmingham, United Kingdom; 5 Birmingham Women’s National Health Service (NHS) Foundation Trust, Birmingham, United Kingdom; The University of Tokyo, JAPAN

## Abstract

**Objective:**

To compare the cost-effectiveness of bladder ultrasonography, clinical history, and urodynamic testing in guiding treatment decisions in a secondary care setting for women failing first line conservative treatment for overactive bladder or urgency-predominant mixed urinary incontinence.

**Design:**

Model-based economic evaluation from a UK National Health Service (NHS) perspective using data from the Bladder Ultrasound Study (BUS) and secondary sources.

**Methods:**

Cost-effectiveness analysis using a decision tree and a 5-year time horizon based on the outcomes of cost per woman successfully treated and cost per Quality-Adjusted Life-Year (QALY). Deterministic and probabilistic sensitivity analyses, and a value of information analysis are also undertaken.

**Results:**

Bladder ultrasonography is more costly and less effective test-treat strategy than clinical history and urodynamics. Treatment on the basis of clinical history alone has an incremental cost-effectiveness ratio (ICER) of £491,100 per woman successfully treated and an ICER of £60,200 per QALY compared with the treatment of all women on the basis of urodynamics. Restricting the use of urodynamics to women with a clinical history of mixed urinary incontinence only is the optimal test-treat strategy on cost-effectiveness grounds with ICERs of £19,500 per woman successfully treated and £12,700 per QALY compared with the treatment of all women based upon urodynamics. Conclusions remained robust to sensitivity analyses, but subject to large uncertainties.

**Conclusions:**

Treatment based upon urodynamics can be seen as a cost-effective strategy, and particularly when targeted at women with clinical history of mixed urinary incontinence only. Further research is needed to resolve current decision uncertainty.

## Introduction

Lower urinary tract symptoms involving urgency or urinary incontinence are prevalent in approximately 20% of the population world-wide [1]. Urinary incontinence is the involuntary leakage of urine, and is classified into urge incontinence, stress incontinence (e.g. with sneezing or coughing), or mixed incontinence—when it is both urge- and stress-related. Overactive bladder is a syndrome characterised by urinary urgency, with or without urge incontinence, and often frequency and nocturia [2]. The prevalence of overactive bladder is estimated in the region of 12–17% of the population [3, 4]. Evidence, however, suggests that it remains a highly underdiagnosed and undertreated syndrome [5]. The cost implications associated with these urinary syndromes and their impact on quality of life have been well documented [6–10].

The uncertainty around the correct diagnosis among syndromes sharing common symptomatology has established urodynamic testing as the gold-standard test when first line conservative treatments have been unsuccessful. Urodynamics provides a pathophysiological explanation of symptoms [11], and lasts approximately 30 minutes. It can identify detrusor overactivity (DO), which commonly is the underlying pathology behind overactive bladder symptoms, or provide alternative diagnoses including urodynamic stress incontinence, mixed incontinence, voiding dysfunction, low compliance, or normal bladder physiology. However, comprehensive evidence on the accuracy of urodynamics is lacking, and its role in determining patient outcomes is increasingly questioned. For patients with uncomplicated stress incontinence, evidence suggests that urodynamics is neither necessary nor cost-effective [12–14]. For patients with overactive bladder, clinical evidence is contradictory. While there are studies concluding that urodynamics is required, as symptoms tend to be an unreliable indicator of DO [15, 16], there are also studies concluding that an urodynamic observation of DO is not a good predictor of the outcome of a number of different treatments [17].

Current guidelines on urinary incontinence recommend conservative management of urinary symptoms as a first line treatment, and the use of urodynamics only prior to more invasive interventions in a secondary care setting [18]. Nevertheless, robust evidence for the use of urodynamics in this context is lacking. The National Institute for Health and Care Excellence (NICE) in the UK has encouraged further investigation into the role of bladder ultrasonography to measure bladder wall thickness, which, if sufficiently accurate, offers a less invasive and potentially cheaper alternative to urodynamics [19]. The objective of the model-based economic evaluation is to compare the relative cost-effectiveness of basing treatment decisions on bladder ultrasonography, clinical history, or urodynamics in women with persistent symptoms of overactive bladder or urge-predominant mixed incontinence for whom first line conservative treatments were not effective.

## Methods

This study reports an economic evaluation carried out alongside the Bladder Ultrasound Study (BUS), the largest cross-sectional study undertaken to estimate the accuracy of ultrasound measurement of bladder wall thickness (BWT) in the diagnosis of DO. Details of the accuracy study are reported in the full *Health Technology Assessment* (HTA) report [20]. In brief, 687 women with symptoms of overactive bladder or urgency-predominant mixed incontinence were recruited across 22 hospitals in the UK. To be included in the study, women had to have urinary frequency of ≥ 9 voids for at least one day in a 3 day bladder diary, mild to severe urgency recorded on at ≥ 2 occasions in the bladder diary, and post void residual volume < 100 ml on the bladder scan to rule out voiding dysfunction. Women with symptoms of pure stress urinary incontinence or stress-predominant mixed incontinence, current pregnancy or up to six weeks postpartum, stress incontinence-related surgery and/or intradetrusor Botulinum toxin A in the past six months, positive urine dipstick for leucocytes or nitrites, pelvic organ prolapse > grade II (any compartment), previous urodynamics in the past six months, and continuous use of antimuscarinics for more than six months were excluded from the study.

Test accuracy was determined by comparing BWT measurements from ultrasonography (index test) against the results obtained from urodynamic testing (reference standard). Urodynamic testing was carried out following the Good Urodynamic Practices Guidelines of the International Continence Society [21]. In the BUS study, DO was defined as the occurrence of involuntary detrusor contractions during filling cystometry that could occur spontaneously or because of provocation [2], and filling cystometry was done with fluid filled domes and fill rate of 100 ml/min in sitting position. Using the ultrasound, BWT was determined as the mean measurement of the thickest part of the trigone, dome of the bladder in the midline, and the anterior wall of the bladder. Sensitivity, specificity and predictive values for ultrasonography were calculated using a BWT threshold of ≥ 5 mm for the diagnosis of DO [22]. All women provided written informed consent and ethical approval was granted from the Nottingham Research Ethics Committee (Reference: 10/H0408/57). Given the objective of the economic evaluation, the analysis relied on data for women who had been taking conservative treatments before enrolling into the study and had complete accuracy data (n = 209), in line with NICE clinical recommendations [18].

### Model structure

For the purpose of maintaining patient history, and given the short-term nature of the decision problem, a decision tree was used to describe the alternative diagnostic options being compared and the treatment pathways determined upon their diagnosis. The model was developed in TreeAge Pro 2014 software (TreeAge Software, Inc., Williamstown, MA, USA) and concerned women with unsuccessful first line conservative treatment. Women in the model would require invasive interventions determined by the findings of urodynamics, bladder ultrasonography, and clinical history. Alternative second line treatment options for each diagnostic finding were modelled based on clinical input and NICE’s guidelines on the management of urinary incontinence [23].

In this study, two different model structures were explored. In the first structure, the three test-treat strategies were directly compared assuming that clinical history plays no role in determining the need for urodynamics or ultrasound, or in influencing treatment options in cases of disagreement. The second structure, allows for these diagnostic tests to depend upon the outcome of clinical history.

**Fig 1:**
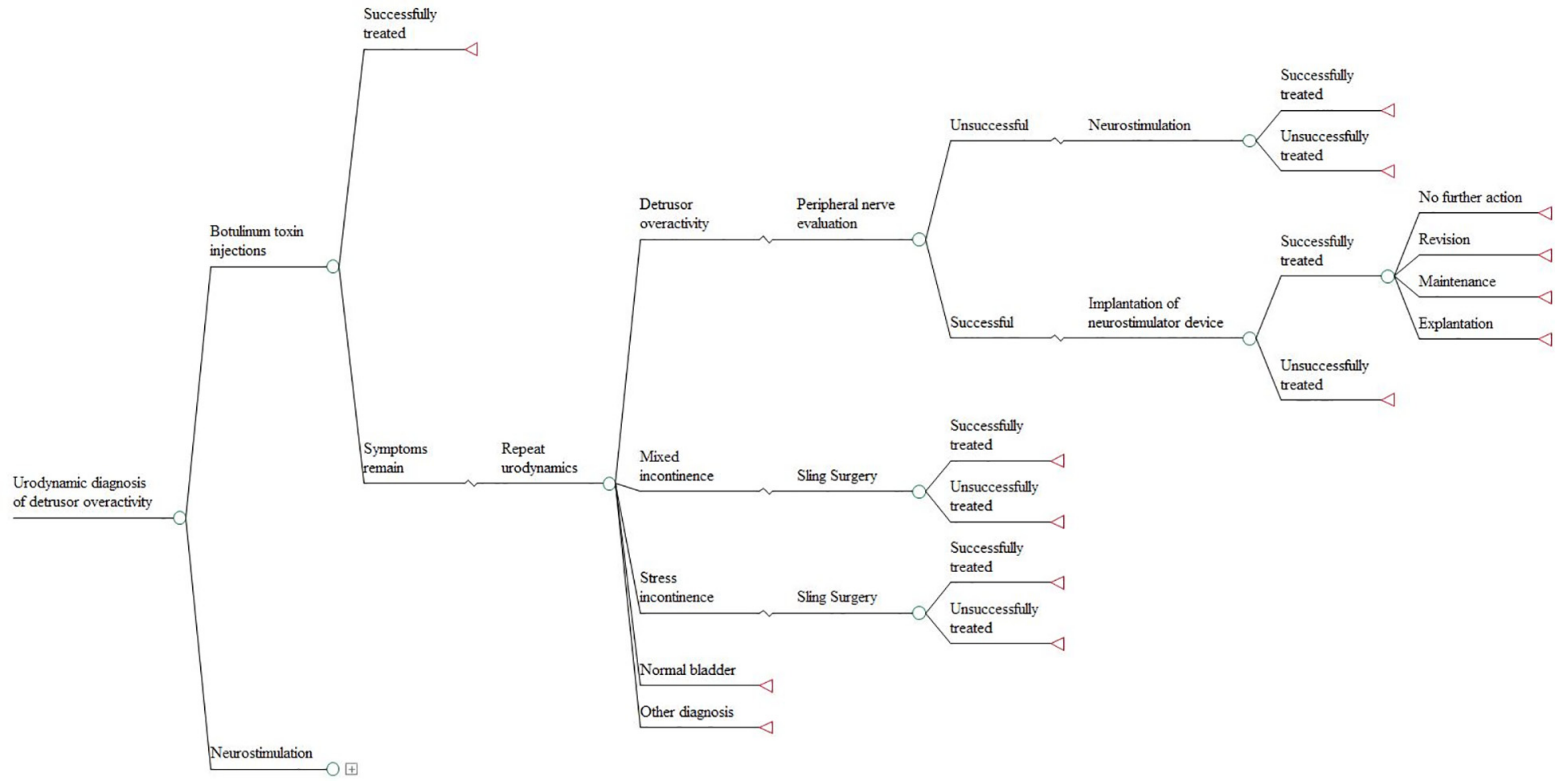
Decision sub-tree representing the treatment pathway of women with an urodynamic diagnosis of detrusor overactivity [The + sign denotes similar model structure].

Urodynamic testing provides diagnoses of overactive bladder, urodynamic stress and mixed incontinence, normal bladder physiology, low compliance, and voiding dysfunction. For overactive bladder, treatment with either botulinum toxin injections or a neurostimulation (percutaneous tibial nerve) was modelled, depending on patient and physician preferences. For women remaining symptomatic, a peripheral nerve evaluation was assumed to follow in order to assess whether a permanent implantation of a neurostimulator device would be successful, or women would require further botulinum toxin injections or neurostimulation, depending on which of the two interventions had not been carried out earlier in the model (Fig 1). For stress incontinence, initial treatment in the model involved a sling surgery followed by a Burch colposuspension if women remained symptomatic, unless treatment for overactive bladder had previously been given. For the treatment of mixed incontinence, a sling surgery and Burch colposuspension were also assumed to take place with the exception that, due to the presence of urinary urgency, women had the option of botulinum toxin injections prior to sling surgery. Women with urodynamic findings of normal bladder physiology, low compliance only, or voiding dysfunction only were assumed to remain symptomatic without further invasive interventions.

Treatment pathways following bladder ultrasonography and clinical history are similar, but reflect variations arising from the fact that for both strategies, treatment for either overactive bladder or mixed urinary incontinence can only be initiated given the urgency-predominant symptoms of the population modelled (S1 and S2 Figs). The primary study assessed the accuracy of bladder ultrasonography for diagnosing DO using a predefined threshold of BWT (5mm) on the basis of previous research [22]. This was followed in the model using accuracy data from the primary study [20]. When BWT is below this threshold, the ultrasound cannot distinguish between alternative urinary syndromes and treatment for mixed incontinence was assumed to be initiated. For clinical history, the criterion of whether women only had urinary urgency, with or without urge incontinence, or had urgency-predominant mixed incontinence was used to indicate diagnoses either of overactive bladder or of mixed incontinence. A more detailed graphical representation of the decision model is provided in the HTA report [20].

### Additional model assumptions

For the purposes of the analysis some further assumptions were required:

- Treatment with botulinum toxin may result in voiding dysfunction [24].
- Women with voiding dysfunction would require self-catheterisation training [25].
- Repeated botulinum toxin injections, commonly provided at yearly intervals, have shown to be clinically effective [26, 27]. It was assumed that women could have up to 3 injections.
- Neurostimulation was offered in 12 sessions, a week apart over the first 3 months [28]. If it was clinically effective, a monthly maintenance session would be needed thereafter [29].
- After an implantation of a neurostimulator device, a revision of the surgery, maintenance or removal of the neurostimulator may be needed [30, 31]. These were assumed to take place at the end of the model’s time horizon.
- A maximum of two diagnostic tests were assumed to be carried out, one when entering the model, and another if women remained symptomatic.
- If a woman became subjectively cured, improvements were assumed to last throughout the model’s time horizon.

### Probabilities

Probabilities for symptoms’ prevalence and test accuracy were taken from the primary study (S1 and S2 Tables) [20]. The probability of a woman becoming subjectively cured by a given intervention and subject to the stated underlying condition, and other probabilities used in the model were drawn from secondary sources [24, 26, 28, 30–37]. These are shown in Table 1. Probabilities applying to rare circumstances, such as in cases of misdiagnosis, were not all available in published sources, and values were, therefore, elicited independently from the expert opinion of eight study collaborators on the 18^th^ of June 2014 (Table 1). In the absence of relevant information, expert opinion is considered a legitimate source of information in decision-analytic modelling [38]. Since these probabilities are not commonly encountered, it was considered appropriate to decide a priori the use of a beta distribution and a simple elicitation approach, asking for most likely value as well as lowest and highest, and interpreting these as mean and 95% confidence intervals (CIs). Use of more elaborate elicitation techniques [39] was not expected to have an appreciable impact on the modelling.

**Table 1:** Effectiveness data and other model parameters.

Parameter	Base-case value₊ (95% CIs)	Distribution (parameter values)‡	References
Probability of subjective cure*			
Botulinum toxin—Detrusor overactivity†	0.568 (0.464, 0.669)ǂ	Beta (50, 38)	Dowson *et al* [26]
Botulinum toxin—Mixed incontinence	0.329 (0.200, 0.483)	Beta (13.42, 27.36)	Expert opinion
Botulinum toxin—Stress incontinence	0.143 (0.133, 0.375)§	Beta (4.32, 25.86)	Expert opinion
Neurostimulation—Detrusor overactivity	0.606 (0.558, 0.653)ǂ	Beta (245, 159)	Burton *et al* [28]
Neurostimulation—Stress/mixed incontinence	0.314 (0.283, 0.567)	Beta (12.36, 27)	Expert opinion
Implanted neurostimulator—Detrusor overactivity	0.675 (0.633, 0.715)ǂ	Beta (338, 163)	Brazzelli *et al* [30]
Implanted neurostimulator—Stress/mixed incontinence	0.271 (0.233, 0.550)	Beta (7.74, 20.82)	Expert opinion
Sling surgery—Detrusor overactivity	0.310 (0.250, 0.350)	Beta (5.84, 13)	Weber and Walters [37]
Sling surgery—Stress incontinence	0.868 (0.841, 0.894)ǂ	Beta (547, 83)	Latthe *et al* [32]
Sling surgery—Mixed incontinence	0.560 (0.534, 0.579)ǂ	Beta (1050, 837)	Jain *et al* [33]
Colposuspension—Detrusor overactivity	0.163 (0.153, 0.386)	Beta (5.95, 30.56)	Expert opinion
Colposuspension—Stress incontinence	0.690 (0.612, 0.762)ǂ	Beta (100, 45)	Dean *et al* [35]
Colposuspension—Mixed incontinence	0.489 (0.381, 0.595)ǂ	Beta (40, 42)	Kulseng‐Hanssen *et al* [36]
***Other probabilities***			
Choose botulinum toxin prior to sling surgery	0.314 (0.275, 0.683)	Beta (5.74, 12.53)	Expert opinion
Choose botulinum toxin over neurostimulation	0.750 (0.421, 0.963)ǂ	Beta (6, 2)	Expert opinion
Voiding difficulties due to botulinum toxin	0.086 (0.042, 0.143)ǂ	Beta (10, 106)	Tincello *et al* [24]
Require implantation of neurostimulator—Detrusor overactivity	0.670 (0.450, 0.880)	Beta (11.23, 5.53)	Brazzelli *et al* [30]
Neurostimulator requires revision < 2 years	0.090 (0.064, 0.119)ǂ	Beta (36, 366)	Siddiqui *et al* [31]
Neurostimulator requires revision ≥ 2 years	0.330 (0.299, 0.362)ǂ	Beta (282, 573)	Brazzelli *et al* [30]
Neurostimulator requires maintenance ≥ 2 years	0.150 (0.111, 0.195ǂ	Beta (42, 237)	Brazzelli *et al* [30]
Neurostimulator requires removal	0.107 (0.068, 0.152ǂǂ	Beta (22, 184)	Siddiqui *et al* [31]

*The figures are probabilities that the patient will be subjectively cured by the given intervention subject to the stated underlying condition. These are given as point estimates with 95% confidence intervals and corresponding beta distributions.

₊ Mean value of a model parameter that was used in main analysis.

† The value was estimated using the disaggregated contribution of each of the three injections (34%, 52%, and 14%) and the subsequent drop-out rates (20%, 8%, and 0%) [26]. The disaggregated contribution and drop-out rates were assumed to be the same across syndromes.

ǂ Information in the source consisted of point estimates of effectively treated number of patients, and 95% confidence intervals (CIs) have been derived from this information.

‡ Where mean and number of patients effectively treated was available, these were used to derive distribution parameters. In other cases, distributions were fitted to the mean and 95% confidence intervals (CIs).

§ This value represents the most conservative estimate of effectiveness in case of misdiagnosis and was used as a proxy of effectiveness in cases where women with low compliance only, voiding dysfunction only, or normal bladder received interventions as a result of misdiagnosis.

### Costs

Cost data were drawn from national sources (Table 2) [40, 41], and calculated in 2012–2013 UK pounds (£). Unit costs adopted from the NHS Reference Costs (2012–13) [40] were calculated based on the weighted average value of elective inpatient and day-case costs and the proportion of patients in each type of care. Unit costs from the “*Urology*” category were selected instead of the average across different medical specialties, apart from the case of the bladder ultrasonography for which a total Healthcare Resource Groups (HRG) cost was only available. The cost of three botulinum toxin injections was calculated by multiplying the unit cost of each injection (£912; 95% CIs: £704–£1,060) by the proportion of women undergoing each injection.

**Table 2:** Unit cost data (£, 2012–13 price base).

Intervention	HRG code*	Base-case value (95% CIs)₊	Distribution (parameter values)‡	References
Urodynamics	LB42A	401 (216–462)	Gamma (40.65, 9.86)	NHS Reference Cost [40]
Bladder ultrasonography	RA23Z†	51	Gamma (1.00, 51.07)	*Ibid*.
Botulinum toxin injection	LB14Z	912 (704–1,060)	Gamma (100.67, 9.06)	*Ibid*.
Neurostimulation	AA21F	2,221 (1,274–2,838)	Gamma (30.81, 72.08)	*Ibid*.
Sling surgery	LB59Z	3,917 (2,599–5,309)	Gamma (31.93, 122.69)	*Ibid*.
Peripheral nerve evaluationǂ	AA21F	1,162 (1,010–1,293)	Gamma (258.88, 4.49)	*Ibid*.
Implantation of neurostimulator	AB07Z	6,530 (4,966–8,347)	Gamma (57.14, 114.28)	*Ibid*.
Removal/ maintenance of neurostimulator	AB04Z	4,160 (2,960–5,831)	Gamma (32.09, 129.65)	*Ibid*.
Burch colposuspension	LB59Z	3,917 (2,599–5,309)	Gamma (31.93, 122.69)	*Ibid*.
Self-catheterisation training§		84	Gamma (1.00, 84.00)	Curtis [41]

* Based on urology category unless otherwise indicated.

₊ Mean value of a model parameter that was used in main analysis.

† Based on total Health Resource Groups (HRG).

ǂ As a day case.

‡ Distributions were fitted based on mean value and 95% confidence intervals (CIs) apart from the cases of bladder ultrasonography and self-catheterisation training, where distributions were fitted by the method of moments assuming a variance equal to the mean cost.

§ Assuming an hour contact with a nurse.

### Outcomes

The primary outcome was *“women successfully treated”*, determined by subjective cure. The secondary outcome was *“quality-adjusted life-years (QALYs)”*, which is the recommended outcome for economic evaluations in the UK [42], and combines quantity with quality of life measured using preference weights. Quality of life data for women entering the model were taken from the primary study (Table 3) [20]. To identify quality of life data for outcomes experienced during the modelled time horizon, a review of the Cost-Effectiveness Analysis Registry [43] was performed using the search terms *“overactive bladder”*, *“urinary incontinence”*, and *“detrusor overactivity”*. Eighteen studies were identified and preference weights from one study were selected to represent the quality of life of women subjectively cured, with and without side-effects [44]. This study was selected because values were obtained using the time trade-off method, which has sound theoretical underpinnings in utility theory, and were relevant to the symptoms and type of interventions modelled [45]. In the absence of other estimates, women remaining symptomatic were assumed to retain their initial quality of life, which is a common assumption in cost-effectiveness analyses in this clinical context [44, 46–48]. QALYs were estimated by combining the utility weights with estimates of duration of the different health states.

**Table 3:** Quality of life data.

Description	Base-case value (95% CIs)₊	Distribution (parameter values)‡	References
Detrusor overactivity	0.600 (0.532–0.668)	Beta (8.96, 5.98)	BUS study [20]
Stress urinary incontinence	0.660 (0.514–0.807)	Beta (18.92, 9.75)	*Ibid*.
Mixed urinary incontinence	0.718 (0.637–0.799)	Beta (49.12, 19.29)	*Ibid*.
Normal bladder	0.656 (0.558–0.753)	Beta (22.13, 11.61)	*Ibid*.
Low compliance or voiding dysfunction	0.744 (0.547–0.942)	Beta (11.76, 4.05)	*Ibid*.
Subjective cure without side effects	0.920 (0.710–0.990)	Beta (10.69, 0.93)	Chen *et al* [44]
Subjective cure with side effects	0.870 (0.830–0.900)	Beta (304, 45.43)	*Ibid*.

₊ Mean value of a model parameter that was used in main analysis.

**‡** Distributions were fitted based on mean value and 95% confidence intervals (CIs).

### Analyses

Two separate economic analyses were carried out from the perspective of the UK National Health Service (NHS) and results were presented in incremental cost-effectiveness ratios (ICERs). The first analysis assessed the cost-effectiveness of basing treatment decision upon bladder ultrasonography and clinical history alone compared with the treatment based upon the gold-standard urodynamics. The second analysis additionally explored all the different ways in which clinical history and a diagnostic test could be used together. This aimed to assess whether a diagnostic test would be more cost-effective when offered in a selective sub-group of women (i.e. those with clinical history of overactive bladder only or those with a clinical history of mixed incontinence only). A 5-year time-horizon was considered appropriate to reflect key differences in terms of costs and benefits for the test-treat strategies compared. Costs and QALYs accruing beyond 12 months were discounted at an annual rate of 3.5% [42].

A number of sensitivity analyses were performed. Univariate analyses explored the impact of: (a) reducing the cost of urodynamics from £401 (95% CIs: £216–£462) to £173, which represents the weighted average value of total HRGs [40]; (b) doubling the cost of sling surgery to account for possible adverse events [32]; and (c) lowering the utility weight of women subjectively cured from 0.92 to 0.84, which represents the mean EQ-5D index score for women who reported low symptoms bother in the primary study [20]. Multivariate analyses explored the impact of: (a) lowering the accuracy of urodynamics [13], while improving the accuracy of clinical history by complementing it with urinary diaries [49]; (b) placing the rates of effectiveness elicited from expert opinion to the lowest and highest value; and (d) assuming one diagnostic test is only performed when entering the model.

For a more comprehensive representation of parameter uncertainty, a probabilistic sensitivity analysis (PSA) was undertaken. Using a Monte Carlo simulation, 10,000 iterations of the model were performed by repeated random draws from probability distributions attached to model parameters. Beta and Dirichlet distributions were used for binomial and multinomial data respectively, and a Gamma distribution for costs [50]. The results from all model iterations were averaged, and at any given level of decision-makers’ willingness-to-pay (WTP) per additional unit of outcome the optimal strategy was determined from the average results. To present the proportion of model iterations that favoured the overall optimal test-treat strategy at each threshold of WTP, cost-effectiveness acceptability frontiers (CEAFs) were used [51]. CEAFs plot the probability of the optimal strategy being cost-effective, under current uncertainty, at different WTP values per additional unit of outcome.

Given that decision uncertainty may lead to sub-optimal policy recommendations, the opportunity cost of gaining further information to assist decision-making in the future needs to be evaluated. A metric which indicates whether undertaking further research is potentially more worthwhile than adopting a decision under current uncertainty is the expected value of perfect information (EVPI). The EVPI represents the maximum monetary value that rational decision-makers would be willing to spend for gaining more information at each threshold of WTP per additional unit of outcome, and combines the probability of a wrong decision with the monetary value of the gain that would be expected in changing the decision from the wrong to the correct one [50, 52, 53]. For example, if the cost of further research, such as a randomised controlled trial, is lower than the EVPI then investing on further research is potentially more worthwhile. The EVPI was estimated at a population level based on estimates of incidence (54,000) [3, 4, 54, 55], and the number of years that research was assumed to be useful (10 years) with 3.5% annual discount rate.

## Results

In the first analysis, a test-treat strategy based upon bladder ultrasonography was dominated by the strategies of urodynamics and clinical history, as it was more expensive and less effective for both outcomes of the analysis. As shown in Table 4, treatment based upon clinical history led to an additional 26 cases per 10,000 women successfully treated compared with treatment based upon urodynamics at an additional cost of £1,278 per woman, and the corresponding ICER was £491,100 per woman successfully treated.

**Table 4:** Table of results.

Test-treat strategy	Cost	Women successfully treated	QALYs	ICER per woman successfully treated	ICER per QALY
***First analysis***					
Urodynamics	£4,524	0.615	3.669		
Clinical history	£5,801	0.618	3.691	£491,100	£60,200
Bladder ultrasonography	£5,947	0.615	3.621	Dominated	Dominated
***Second analysis***					
Urodynamics—All women	£4,524	0.615	3.669		
Urodynamics—Conditional on clinical history of mixed incontinence	£5,126	0.646	3.717	£19,500	£12,700
Urodynamics—Conditional on clinical history of overactive bladder	£5,198	0.587	3.643	Dominated	Dominated
Bladder ultrasonography—Conditional on clinical history of mixed incontinence	£5,768	0.654	3.689	£78,600	Dominated
Clinical history—All women	£5,801	0.618	3.691	Dominated	Dominated
Bladder ultrasonography—All women	£5,947	0.615	3.621	Dominated	Dominated
Bladder ultrasonography—Conditional on clinical history of overactive bladder	£5,965	0.596	3.636	Dominated	Dominated

QALY, quality-adjusted life-year; ICER, incremental cost-effectiveness ratio.

**Fig 2:**
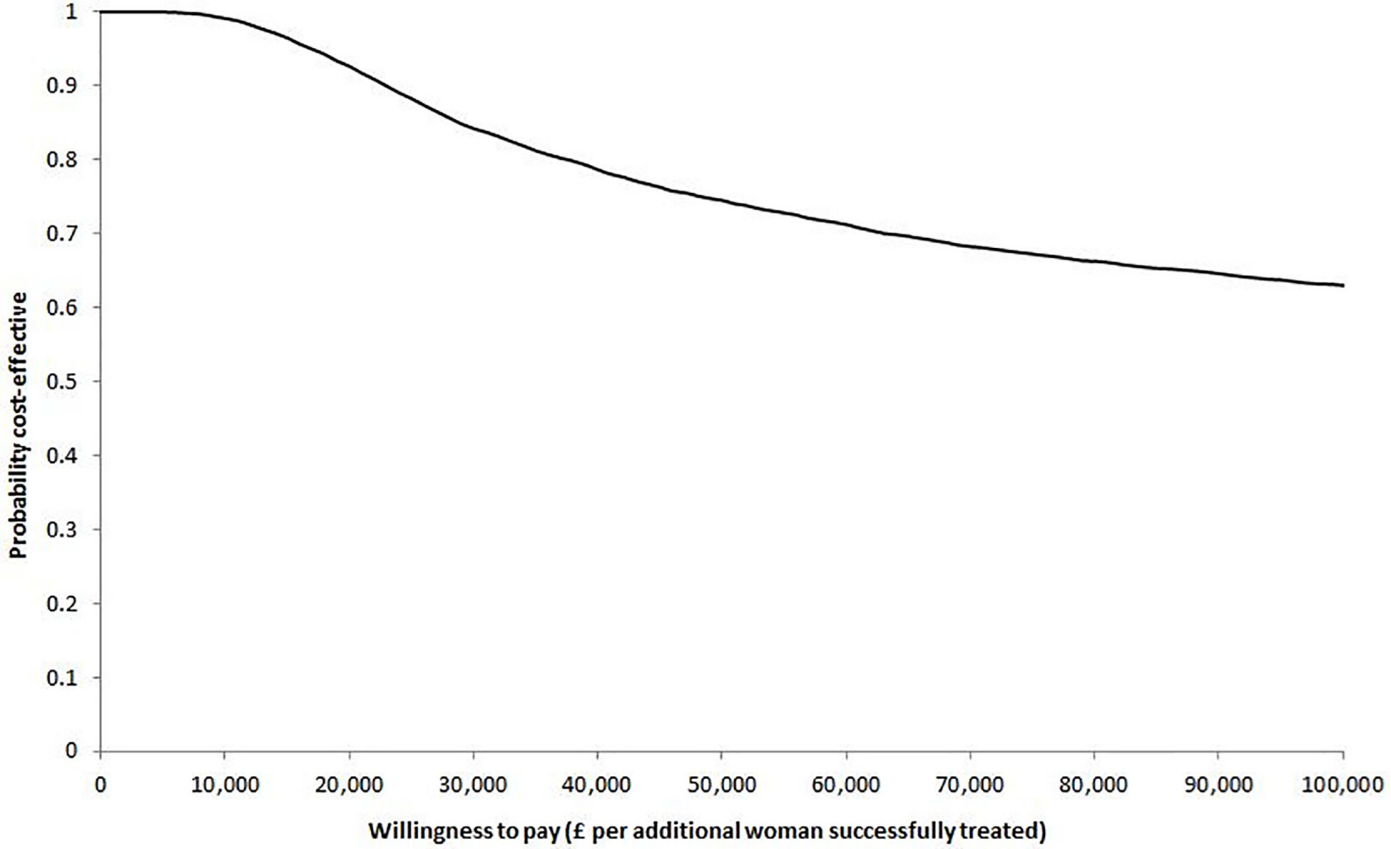
Cost-effectiveness acceptability frontier (CEAF) indicating the probability of the optimal test-treat strategy (i.e. urodynamics) being cost-effective across different willingness to pay thresholds per additional woman successfully treated.

**Fig 3:**
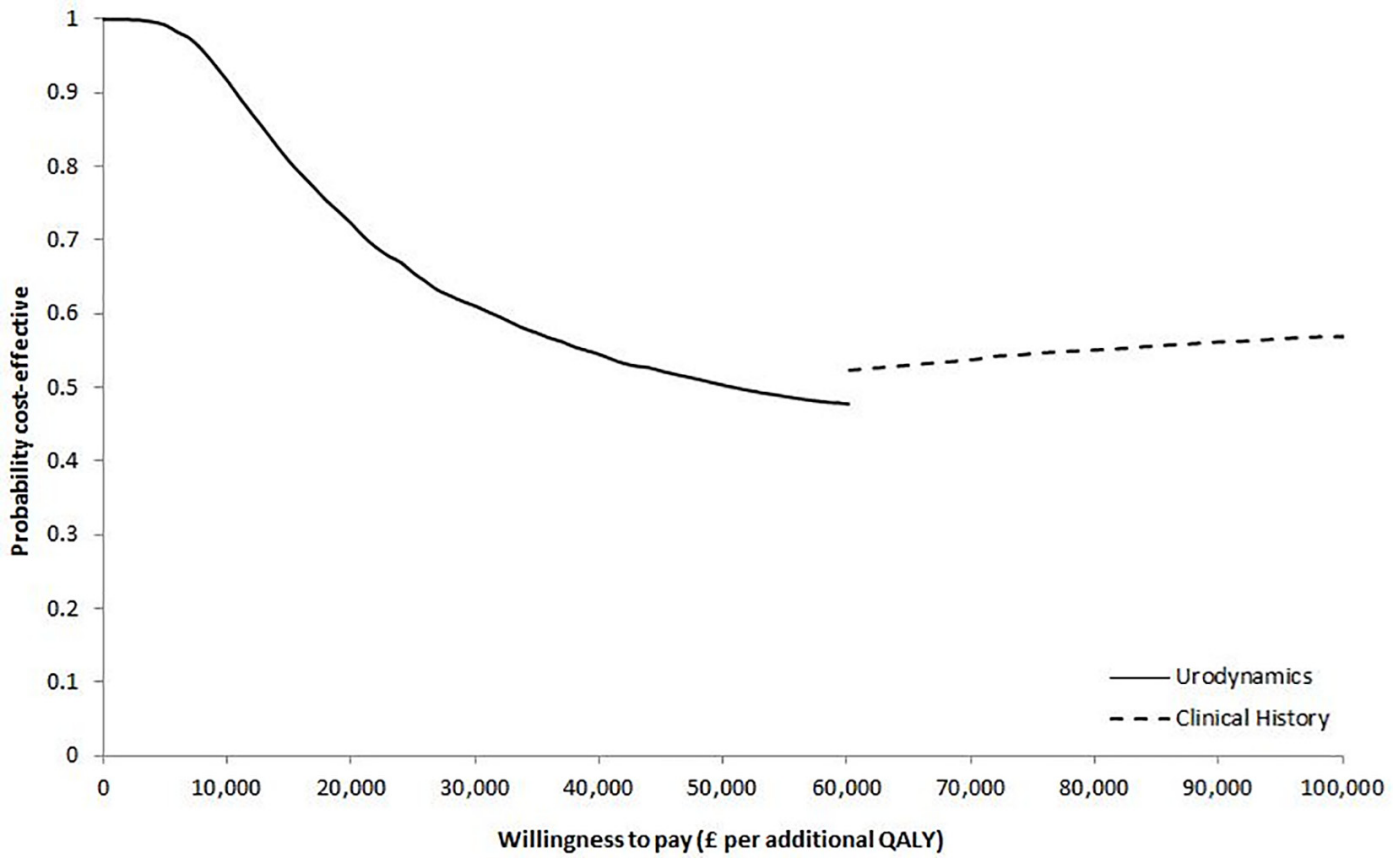
Cost-effectiveness acceptability frontier (CEAF) indicating the probability of the optimal test-treat strategy being cost-effective across different willingness to pay thresholds per additional quality-adjusted life-year (QALY).

The CEAF in Fig 2 shows that the probability of the urodynamics test-treat strategy being cost-effective exceeded 61% for any value of WTP below £100,000 per additional woman successfully treated. In terms of QALYs, treatment based upon clinical history resulted in an additional 0.021 QALYs gained per woman compared with treatment based upon urodynamics. Given the additional cost of £1,278 per woman, the mean ICER for the clinical history strategy compared with urodynamics was estimated at £60,200 per QALY. The results of the PSA show that urodynamics was likely to be the optimal test-treat strategy with 72% probability of being cost-effective at the conventional threshold of £20,000 per QALY (Fig 3) [56].

**Fig 4:**
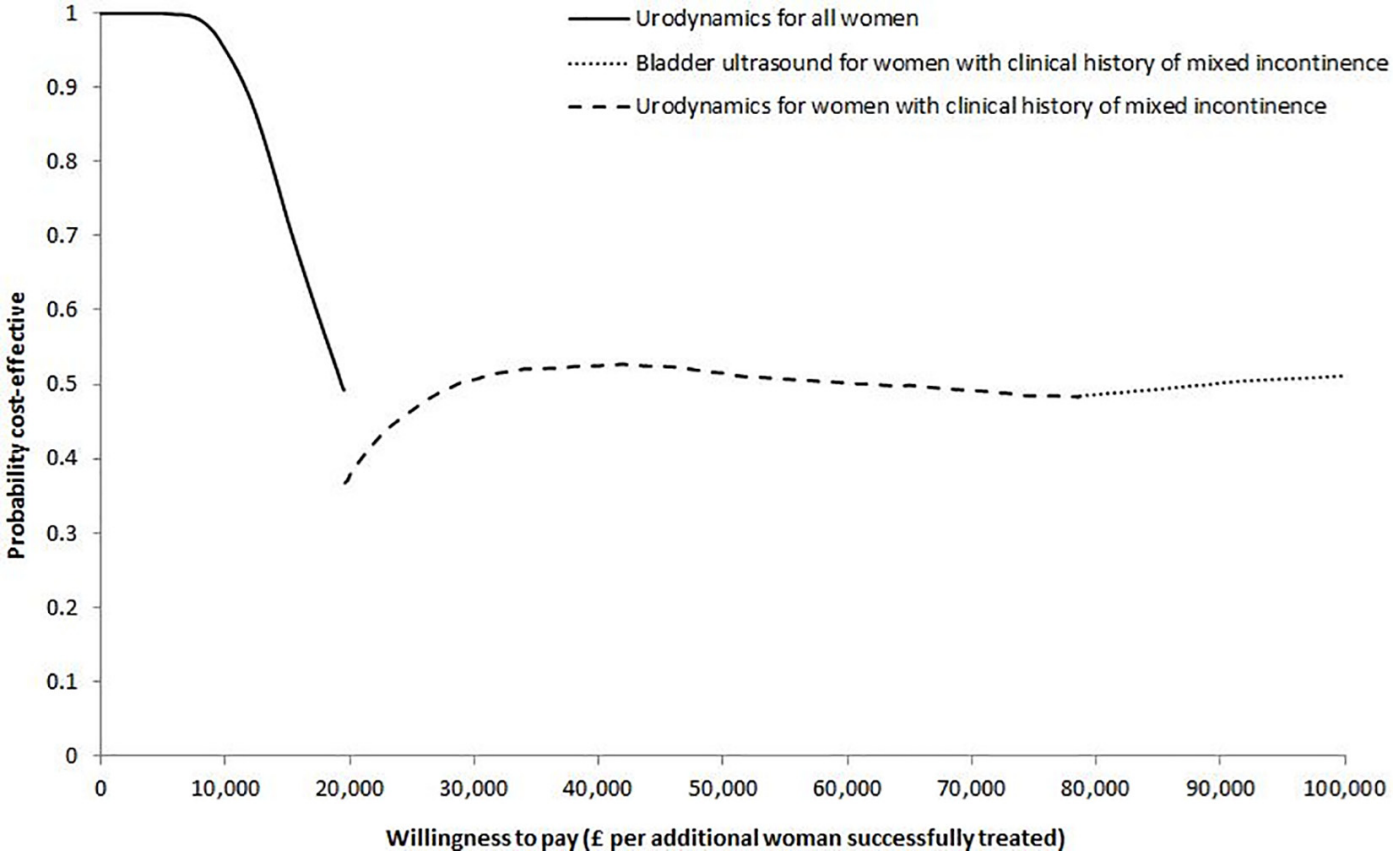
Cost-effectiveness acceptability frontier (CEAF) indicating the probability of the optimal test-treat strategy being cost-effective across different willingness to pay thresholds per additional woman successfully treated.

The results of the second analysis showed that, in comparison to the treatment of all women on the basis of urodynamics, restricting the use of urodynamics to women with clinical history of mixed urinary incontinence led to an additional 309 successfully treated women per 10,000 at an additional cost of £603 per woman (Table 4). This resulted in an ICER of £19,500 per woman successfully treated. Treating these women on the basis of bladder ultrasonography required an additional £641 per woman and led to an additional 82 cases per 10,000 women successfully treated compared with the use of urodynamics in the same women, which gave an ICER of £78,600 per additional woman successfully treated. In the absence of a pre-specified threshold of WTP per additional woman successfully treated, the identification of the probability of the optimal strategy being cost-effective is less straightforward, but £28,000 could be a theoretically acceptable threshold of WTP per woman subjectively cured [20]. At this value, there is almost 50% probability that performing urodynamics only in women with a clinical history of mixed incontinence is a cost-effective test-treat strategy (Fig 4).

**Fig 5:**
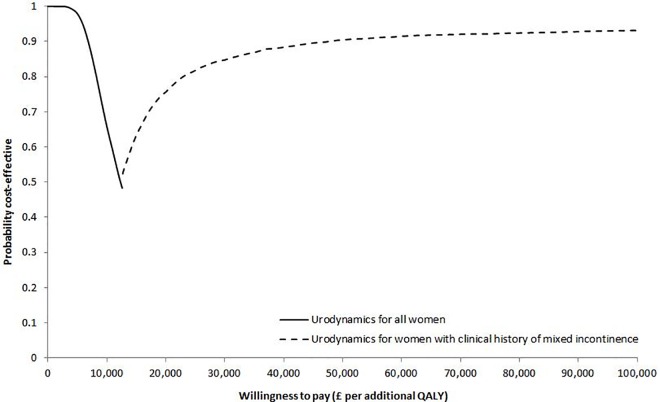
Cost-effectiveness acceptability frontier (CEAF) indicating the probability of the optimal test-treat strategy being cost-effective across different willingness to pay thresholds per additional quality-adjusted life-year (QALY).

In terms of QALYs, having urodynamics as an adjunct to clinical history in women with patient history of mixed incontinence led to an additional 0.476 QALYs gained per woman at an additional cost of £603 compared with the treatment of all women on the basis of urodynamics, leading to an ICER of £12,700 per additional QALY. The results of the PSA indicated that, under current uncertainty, treating only in women with clinical history of mixed incontinence based upon urodynamics, with the rest being treated based upon clinical history, was likely to be the optimal test-treat strategy with 76% probability of being cost-effective at the commonly cited threshold of £20,000 per QALY (Fig 5). Conclusions drawn from both analyses remained robust to all sensitivity analyses except for the scenario where the highest effectiveness rates elicited from expert opinion were assumed in all cases of misdiagnosis (S3 Table).

**Fig 6:**
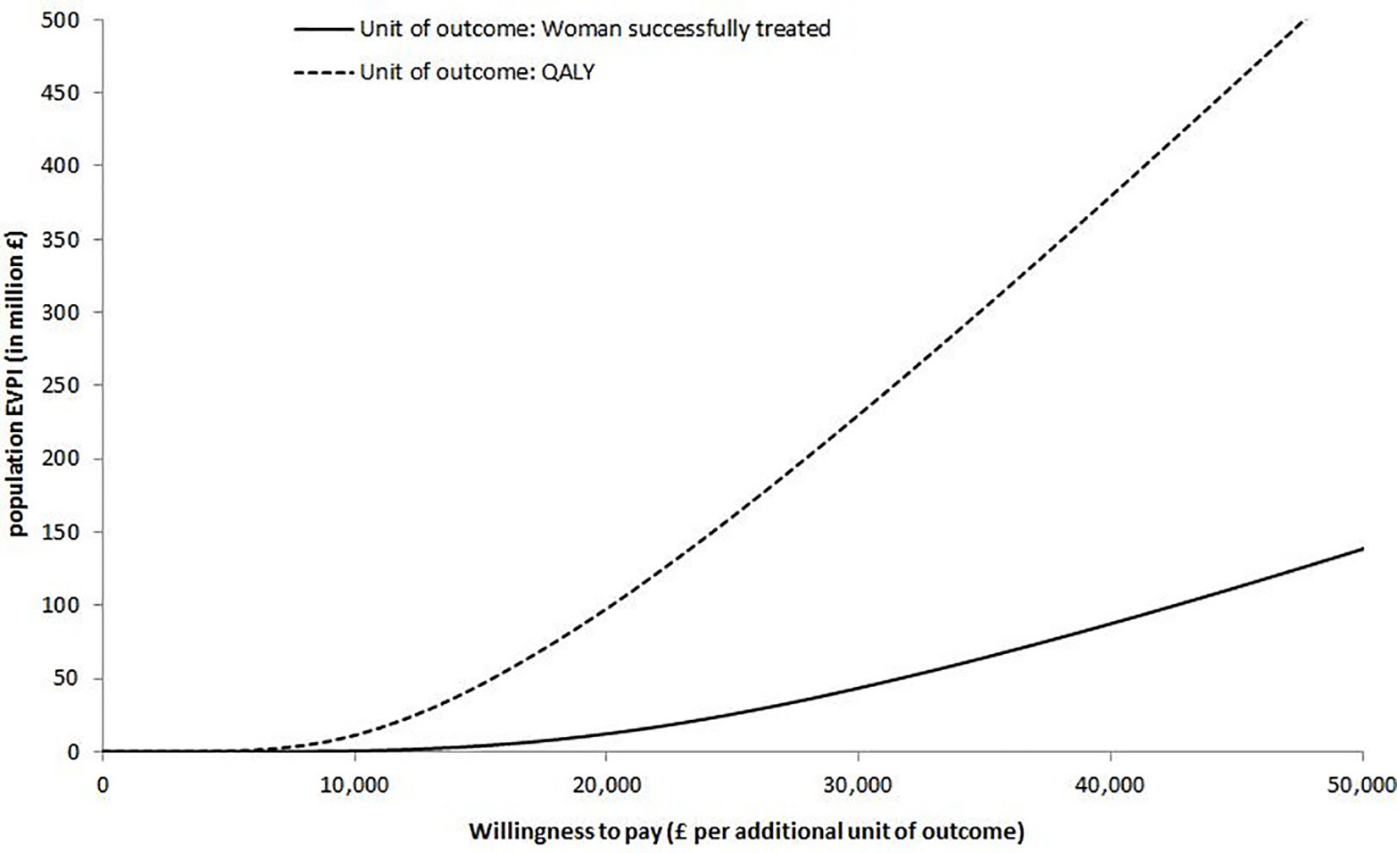
Population expected value of perfect information (EVPI) for the two outcomes of first analysis.

**Fig 7:**
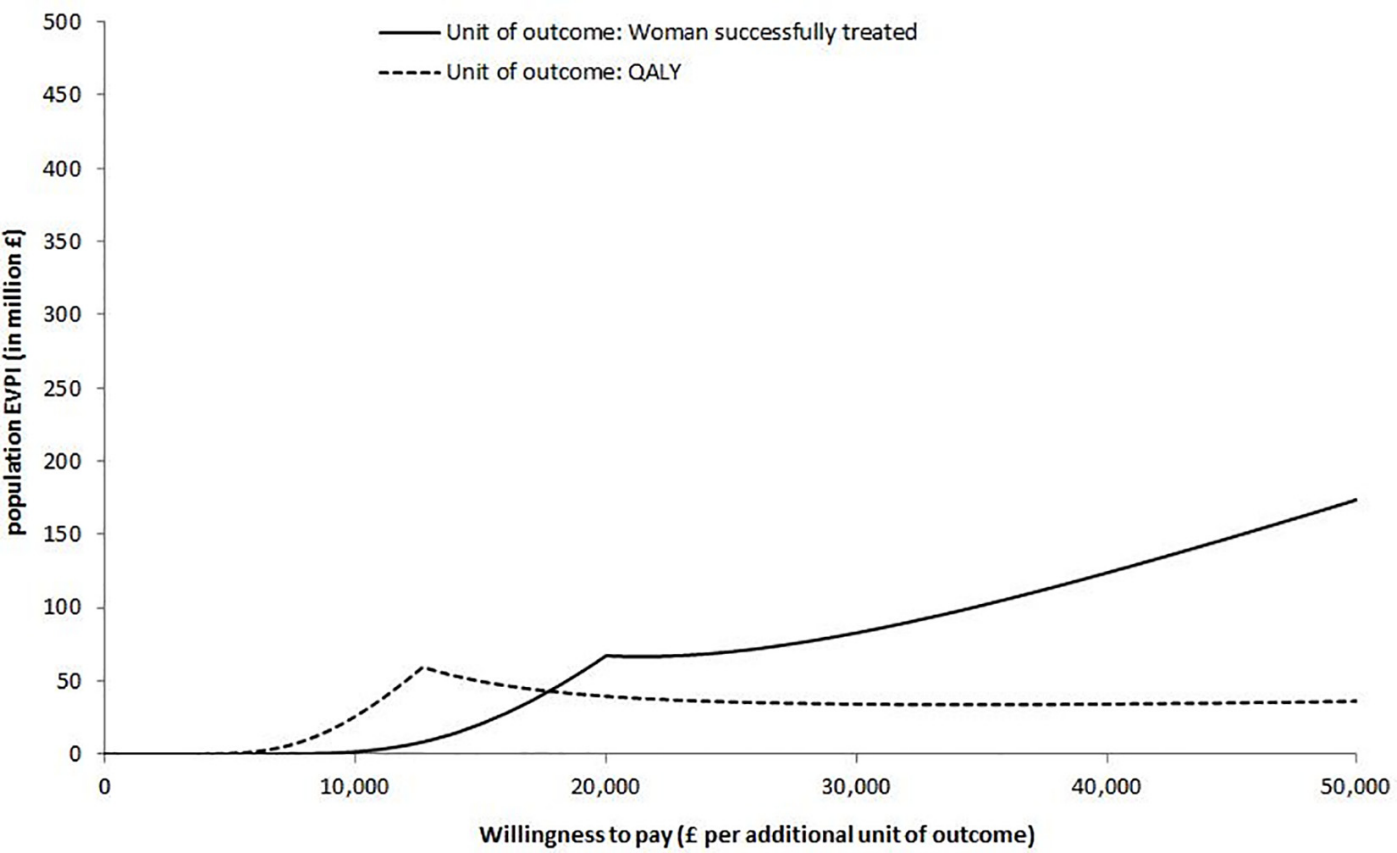
Population expected value of perfect information (EVPI) for the two outcomes of second analysis [The “spikes” in the figure correspond to the points where decision changes, namely to the ICER of the test-treat strategy “Urodynamics for women with clinical history of mixed incontinence” compared with “Urodynamics for all women”, which was £19,500 per woman successfully treated and £12,700 per QALY].

Figs 6 and 7 show the population expected value of perfect information (EVPI) for both outcomes (i.e. women successfully treated and QALYs) of the two analyses undertaken across a range of WTP values for an additional unit of outcome. For the thresholds of £28,000 per woman successfully treated and £20,000 per QALY, population EVPI was found to be £36 million and £98 million respectively for the first analysis, and £77 million and £40 million for the second analysis. The large population EVPI indicates that further research for reducing current decision uncertainty is worthwhile.

## Discussion

### Main findings

This model-based economic evaluation suggested that urodynamics is a cost-effective component in the treatment of women failing first line conservative treatments. The first analysis showed that whilst treating on the basis of clinical history resulted in slightly more cases of women successfully treated and QALYs over the modelled time period compared with urodynamics, treating on the basis of urodynamics led to a worthwhile saving in cost achieved by the lower levels of unnecessary and expensive treatment. The second analysis explored whether urodynamic testing of a sub-group of women would provide a more efficient use of public health care resources. The analysis concluded that the more cost-effective test-treat strategy was to treat women with clinical history of overactive bladder without diagnostic testing and to undertake urodynamics in women with clinical history of mixed urinary incontinence. For both analyses, decisions remained robust to extensive sensitivity analysis concerning the model assumptions but subject to large uncertainties relating to the data inputs, which, as shown in the value-of-information analysis, indicate that further primary research is required.

### Strengths and limitations

To the best of our knowledge, this is the first study attempting to compare the cost-effectiveness of urodynamics, bladder ultrasonography, and clinical history as test-treat strategies for women with symptoms of overactive bladder or urgency-predominant mixed incontinence referred to a secondary care setting. The study benefited from prevalence and accuracy data directly estimated from the BUS study. Costs were informed from national sources, and the main clinical pathways were parameterised with data mostly from meta-analyses and systematic reviews, and based on national treatment guidelines. These are likely to have extended the generalisability of the study’s findings beyond the UK setting. Finally, all assumptions used in the model were agreed in advance and key assumptions were tested in sensitivity analyses.

There are, however, a number of limitations. The results of the economic evaluation may overstate the cost-effectiveness of urodynamic testing. On one hand, this is because urinary diaries complement clinical history in practice, and thus providing a joint decision based upon clinical assessment rather than patient history alone. On the other hand, ambulatory urodynamics may be more sensitive in detecting DO [57], and other diagnoses [58, 59], than conventional laboratory urodynamics. An attempt to explore the cost-effectiveness of the different strategies in light of this evidence was undertaken in a sensitivity analysis. Furthermore, in the absence of available estimates of clinical effectiveness in cases of misdiagnosis, values were elicited from expert opinion. Deterministic and probabilistic sensitivity analyses were undertaken to account for the uncertainty around these estimates. Another limitation is that symptoms of urinary incontinence are commonly associated with profound personal costs. Therefore, economic analyses from a societal perspective may provide further insight into the cost-effectiveness of the test-treat strategies modelled.

An additional limitation relates to the quality of life data used in the model. In the absence of robust utility weights, it was assumed that women remaining symptomatic would maintain their initial quality of life, or that they would have a utility score of 0.92 if they became subjectively cured, with the impact of an alternative utility score being explored in a sensitivity analysis. In reality, however, utility decrements can occur after invasive interventions, and utility gains may differ among different urinary syndromes or severity of symptoms. This lack of evidence is potentially attributable to the common use of disease-specific outcome measures, which offer limited usefulness as outcome measures in cost-effectiveness analyses [60], although mapping algorithms have now started to be developed [61]. Nevertheless, lower urinary tract symptoms are likely to be associated with significant non-health impacts (e.g. embarrassment) and instruments capturing wider wellbeing benefits, such as the ICECAP measures, may offer a more relevant evaluative framework for treatment outcomes [62].

Finally, although the model’s time horizon is long enough to capture important costs and outcomes associated with the different test-treat strategies, the assumption that subjective improvements will last for the modelled period may not be completely valid in all instances. For example, tachyphylaxis to neurostimulation is not well known over time. It is better studied in botulinum toxin injections where there is a known tachyphylaxis rate with repeated injections, although this is small. Such variations from the model assumption, however, were not expected to have a significant impact on the study’s findings.

### Interpretation

Based on the decision model used and the limitations discussed, the results of the first analysis indicated that determining second line invasive interventions in women with persistent symptoms of overactive bladder or urgency-predominant mixed incontinence on the basis of urodynamics is the optimal strategy. This finding supports the conclusions of other studies in favour of urodynamic testing [15, 16]. These studies, however, are all underpinned by the premise that effective treatment for overactive bladder symptoms is only determined through a successful identification of DO, symptom which explains a part of overactive bladder syndrome, and which can only be identified urodynamically. This study also used urodynamics as a gold-standard test, and this is a potential limitation because its accuracy is still open to debate.

An increasing number of studies conclude that an urodynamic identification of DO does not predetermine the clinical effectiveness of a number of different interventions for women with overactive bladder [17, 63–65]. These findings are aligned to the conclusions drawn from the second analysis, which indicated that the most cost-effective test-treat strategy is to carry out urodynamic testing only in women with a clinical history of mixed urinary incontinence. Restricting the use of urodynamics to these women seems to be further supported from evidence pointing out that mixed urinary symptoms are more commonly encountered than mixed conditions [66], which result in a large proportion of women reporting symptoms of mixed urinary incontinence in clinical history [67]. The order of magnitude of this figure appears to be around 50% [66], which is similar to what was found in the BUS study (52%) [20].

### Conclusion

The findings of this paper indicate that restricting the use of urodynamics to women with patient history of mixed urinary incontinence only is potentially the more cost-effective way forward for guiding second line treatment decisions. Further research is, however, needed to reduce the expected opportunity loss associated with the decision of moving away from current practice of basing invasive treatments for all women failing first line conservative treatments on urodynamic findings.

## Supporting Information

S1 Fig Decision sub-tree representing the treatment pathway of women with a diagnosis of detrusor overactivity from bladder ultrasonography; The [+] sign denotes similar model structure. (TIF)

S2 Fig Decision sub-tree representing the treatment pathway of women with a diagnosis of overactive bladder from clinical history; The [+] sign denotes similar model structure. (TIF)

S1 Table Prevalence data. (PDF)

S2 Table Accuracy data. (PDF)

S3 Table Results of univariate and multivariate sensitivity analyses. (PDF)
