# Supplementary figures and images for: Testing and Treating Women after Unsuccessful Conservative Treatments for Overactive Bladder or Mixed Urinary Incontinence: A Model-Based Economic Evaluation Based on the BUS Study
Source: PLoS One. 2016 Aug 11;11(8):e0160351. doi: 10.1371/journal.pone.0160351 (PMC4981306; doi:10.1371/journal.pone.0160351)

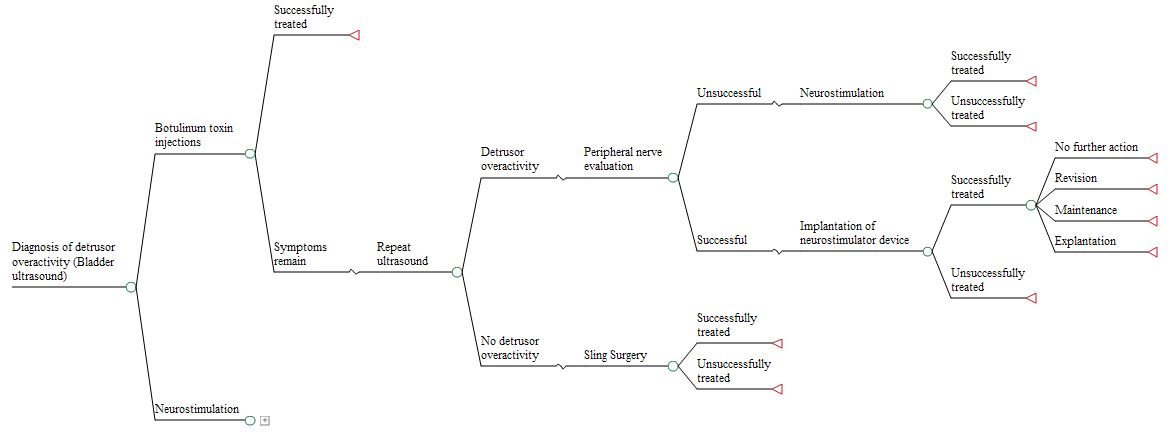

Supplement: S1 Fig — (TIF) [file pone.0160351.s001.tif]

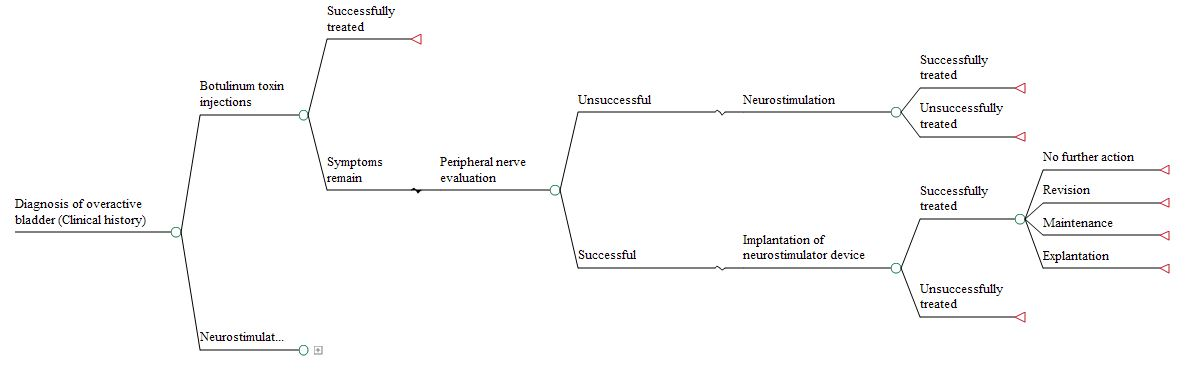

Supplement: S2 Fig — (TIF) [file pone.0160351.s002.tif]
