# Supplementary material for: Testing and Treating Women after Unsuccessful Conservative Treatments for Overactive Bladder or Mixed Urinary Incontinence: A Model-Based Economic Evaluation Based on the BUS Study
Source: PLoS One. 2016 Aug 11;11(8):e0160351. doi: 10.1371/journal.pone.0160351 (PMC4981306; doi:10.1371/journal.pone.0160351)
Supplement: S1 Table — (PDF) [file pone.0160351.s003.pdf]

**S1 Table.** Prevalence data

| Parameter | Base-case value | Distribution (parameter values) |
|---|---|---|
| Detrusor overactivity | 0.507 | Dirichlet (106, 19, 35, 36, 9, 3, 1) |
| Stress incontinence | 0.091 | |
| Mixed incontinence | 0.167 | |
| Normal bladder | 0.172 | |
| Low compliance only | 0.043 | |
| Voiding dysfunction only | 0.014 | |
| Low compliance and voiding dysfunction | 0.005 | |
