# Supplementary material for: Testing and Treating Women after Unsuccessful Conservative Treatments for Overactive Bladder or Mixed Urinary Incontinence: A Model-Based Economic Evaluation Based on the BUS Study
Source: PLoS One. 2016 Aug 11;11(8):e0160351. doi: 10.1371/journal.pone.0160351 (PMC4981306; doi:10.1371/journal.pone.0160351)
Supplement: S2 Table — (PDF) [file pone.0160351.s004.pdf]

S2 Table. Accuracy data

Parameter*	Base-case value	Distribution (parameter values)
Urodynamics		
Diagnosis of detrusor overactivity given detrusor overactivity	0.964	Dirichlet (107, 1, 1, 1, 1)
Diagnosis of stress incontinence given detrusor overactivity	0.009	
Diagnosis of mixed incontinence given detrusor overactivity	0.009	
Diagnosis of normal bladder given detrusor overactivity	0.009	
Diagnosis of other diagnosis given detrusor overactivity	0.009	
Diagnosis of stress incontinence given stress incontinence	0.833	Dirichlet (20, 1, 1, 1, 1)
Diagnosis of mixed incontinence given stress incontinence	0.042	
Diagnosis of detrusor overactivity given stress incontinence	0.042	
Diagnosis of normal bladder given stress incontinence	0.042	
Diagnosis of other diagnosis given stress incontinence	0.042	
Diagnosis of mixed incontinence given mixed incontinence	0.900	Dirichlet (36, 1, 1, 1, 1)
Diagnosis of stress incontinence given mixed incontinence	0.025	
Diagnosis of detrusor overactivity given mixed incontinence	0.025	
Diagnosis of normal bladder given mixed incontinence	0.025	
Diagnosis of other diagnosis given mixed incontinence	0.025	
Bladder ultrasonography†		
Diagnosis of detrusor overactivity given detrusor overactivity	0.377	Beta (40, 66)
Diagnosis of detrusor overactivity given mixed incontinence	0.514	Beta (18, 17)
Diagnosis of detrusor overactivity given stress incontinence	0.474	Beta (9, 10)
Diagnosis of detrusor overactivity given normal bladder	0.444	Beta (16, 20)
Diagnosis of detrusor overactivity given other syndrome	0.462	Beta (6, 7)
Clinical history‡		
Diagnosis of overactive bladder given detrusor overactivity	0.481	Beta (51, 55)
Diagnosis of overactive bladder given mixed incontinence	0.114	Beta (4, 31)
Diagnosis of overactive bladder given stress incontinence	0.105	Beta (2, 17)
Diagnosis of overactive bladder given normal bladder	0.500	Beta (18, 18)
Diagnosis of overactive bladder given other syndrome	0.692	Beta (9, 4)

*When a single diagnostic test is used to determine the presence or absence of a single condition, it is usual to express the test accuracy in terms of sensitivity and specificity. However, when there are more possibilities than simply “disease positive/negative” and “test positive/negative”, these terms cease to be helpful. Instead, it is appropriate to give the conditional probability of a given test result subject to a given actual underlying condition.

† Bladder ultrasonography is used to measure bladder wall thickness (BWT). If BWT is ≥5mm we assume that the test is positive for detrusor overactivity and negative otherwise

‡ Detrusor overactivity (DO) is an urodynamic observation, and thus overactive bladder is used to predict DO and subsequently the right treatment pathway. Women with DO, or other urodynamic diagnoses, who did not have a clinical history of overactive bladder had a clinical history of mixed incontinence
