# Supplementary material for: Testing and Treating Women after Unsuccessful Conservative Treatments for Overactive Bladder or Mixed Urinary Incontinence: A Model-Based Economic Evaluation Based on the BUS Study
Source: PLoS One. 2016 Aug 11;11(8):e0160351. doi: 10.1371/journal.pone.0160351 (PMC4981306; doi:10.1371/journal.pone.0160351)
Supplement: S3 Table — (PDF) [file pone.0160351.s005.pdf]

S3 Table. Results of univariate and multivariate sensitivity analyses

Test-treat strategy	ICER per woman successfully treated	ICER per QALY
Reducing the cost of urodynamics (first analysis)		
Urodynamics		
Clinical history	£602,800	£73,900
Reducing the cost of urodynamics (second analysis)		
Urodynamics for all women		
Urodynamics for women with clinical history of mixed incontinence	£23,200	£15,100
Bladder ultrasonography for women with clinical history of mixed incontinence	£100,000	Dominated
Increasing the cost of sling surgery (first analysis)		
Urodynamics		
Clinical history	£706,800	£86,600
Increasing the cost of sling surgery (second analysis)		
Urodynamics for all women		
Urodynamics for women with clinical history of mixed incontinence	£17,600	£11,400
Bladder ultrasonography for women with clinical history of mixed incontinence	£119,100	Dominated
Lowering the utility of women subjectively cured (first analysis)		
Urodynamics		
Clinical history	£491,100	Dominated
Lowering the utility of women subjectively cured (second analysis)		
Urodynamics for all women		
Urodynamics for women with clinical history of mixed incontinence	£19,500	£22,500
Bladder ultrasonography for women with clinical history of mixed incontinence	£78,600	Dominated
Altering the diagnostic accuracy of urodynamics and clinical history (first analysis)		
Urodynamics		
Clinical history	£63,700	£31,000
Altering the diagnostic accuracy of urodynamics and clinical history (second analysis)		
Urodynamics for all women		
Urodynamics for women with clinical history of mixed incontinence	£16,500	£10,500
Bladder ultrasonography for women with clinical history of mixed incontinence	£39,600	Dominated
Placing elicited values to the lowest limit (first analysis)		
Urodynamics		
Clinical history	Dominated	£66,900
Placing elicited values to the lowest limit (second analysis)		
Urodynamics for all women		
Urodynamics for women with clinical history of mixed incontinence	£20,900	£13,100
Placing elicited values to the highest limit (first analysis)		
Urodynamics		
Clinical history	£30,700	£21,000
BUS	£34,800	Dominated
Placing elicited values to the highest limit (second analysis)		
Urodynamics for all women		
Bladder ultrasonography for women with clinical history of mixed incontinence	£14,000	Dominated
Urodynamics for women with clinical history of mixed incontinence	Dominated	£11,500
Clinical history	Dominated	£83,800
Conducting one diagnostic test (first analysis)		
Urodynamics		
Clinical history	£555,000	£69,500
Conducting one diagnostic test (second analysis)		
Urodynamics for all women		
Urodynamics for women with clinical history of mixed incontinence	£22,300	£14,400

ICER, incremental cost-effectiveness ratio; QALY, quality-adjusted life-year; CI, confidence interval
